# Emerging downhole sensing technologies for mineral exploration

**DOI:** 10.1007/s44346-026-00023-w

**Published:** 2026-11-06

**Authors:** Lesego Senjoba, Matthew Eyre, Rich Crane, Matthew Tonkins, Frances Wall, Kevin Mallin

**Affiliations:** 1 Critical Minerals Challenge Centre, Camborne School of Mines, University of Exeter, Penryn Campus, Penryn, TR10 9FE UK; 2 Geolorn Limited, Stirling, FK8 1NE Scotland, UK

**Keywords:** Mineral exploration, Downhole sensing, Drilling technologies, Data-driven exploration, Subsurface characterization

## Abstract

Recent advancements in spectroscopic, imaging, and geophysical sensors present new opportunities to gather geological information directly during drilling, potentially reducing reliance on physical core samples. These technologies have the potential to reduce drilling costs, shorten exploration timelines, and improve decision-making in early-stage mineral exploration. This review evaluates the principles, capabilities, and limitations of emerging sensing technologies applied to mineral exploration, including hyperspectral imaging, X-ray fluorescence (XRF), gamma-ray spectrometry (GRS), borehole radar (GPR), borehole transient electromagnetic method (BTEM), muon tomography, and seismic-while-drilling (SWD). Key metrics considered include detection limits, spatial resolution, and technology readiness level. Although many of these tools are well established, their integration into drilling systems is constrained by the challenging operating conditions, including narrow borehole dimensions, high vibration, high temperatures and difficulties in maintaining reliable telemetry. By comparing the technological maturity, environmental resilience, and engineering requirements of these sensors, this review identifies GRS, and SWD as having strong potential for near-term deployment during drilling, while XRF, GPR and BTEM are likely to have a transformative impact in the longer term. Realising the full potential of downhole sensing will require substantive advances and investment in sensor miniaturization, ruggedisation for high-pressure and high-temperature environments, along with the development of reliable telemetry systems for real-time data transmission. Additionally large-scale field demonstrations will also be critical for validating system performance and enabling broader industry adoption. These advancements have the potential to transform mineral exploration by improving targeting efficiency, reducing geological uncertainty, and accelerating the transition from discovery to resource development.

## Introduction

For decades obtaining drill cores has been the foundation of subsurface characterization, as they offer direct evidence of lithology, mineralogy, and structure, forming the basis of our understanding of the subsurface [1]. However, as near-surface deposits become depleted, mineral exploration is increasingly shifting focus to deeper and more complex ore bodies. In such cases, traditional coring methods are increasingly slower, expensive and exhibit greater environmental impact compared to alternative drilling techniques. This is largely due to the increasing drilling depth, varying rock conditions, and the need for denser drilling to address complex mineralization. Moreover, drillholes provide predominantly one-dimensional information along the borehole; therefore, multiple drillholes are required to adequately characterize the three-dimensional variability of complex ore bodies. For example, porphyry copper deposits often require deep and closely spaced drilling to accurately capture the spatial variability of grade and alteration. In such settings, drilling serves two critical functions: It aims not only to intersect mineralization but also to gather detailed geological and geochemical data that help interpret alteration zonation and guide exploration toward ore. Additionally, the ongoing need to analyse subtle indicators, such as veining intensity and alteration patterns, further increases the time and cost associated with exploration [2]. To overcome this, advances in high-resolution downhole imaging, spectrometry, and geophysical sensor technologies could play an increasingly important role in subsurface characterization, potentially saving significant time and resources, accelerating on-site decision-making, and reducing reliance on conventional core sampling.

Despite this emerging potential, the mineral industry continues to rely heavily on core samples as the accepted standard for subsurface characterization in exploration. Regulatory frameworks like NI 43-101 and the JORC Code require verifiable evidence of geological features, alteration, and mineralization in reporting [3]. This evidence is often supported by drilling data. Although diamond drill core provides the most detailed geological information, other methods such as reverse circulation (RC) and rotary air blast (RAB) drilling, also provide valuable data for regulatory reporting. However, diamond core drilling is a relatively slow, and resource-intensive process. According to GreenPeg [4] drilling has a significantly greater environmental footprint compared to other exploration techniques such as geophysical surveys. A single drilling campaign may extend over several weeks to months and encompasses not only the drilling process itself but also the transportation of equipment, core logging, sample collection, laboratory analysis, and the long-term storage of core materials [5, 6]. Although these steps are essential for understanding geological features, they require significant time and resources. Drilling and the associated assays often represent the most costly phase of mineral exploration [7]. According to Atlas CopCo [8], drilling usually represents about half of the total exploration budget. However, the actual time spent on drilling constitutes typically only about one-third of the time required to recover a full core barrel. The remaining time is dedicated to ancillary tasks, including pumping fluids, handling drill rods, and retrieving cores. Additionally, core recovery is often hindered by practical challenges such as core loss, jamming, and breakage, all of which can negatively impact the recovery rate [9]. Core recovery is ultimately governed by three key factors: time, cost, and confidence. Although diamond drilling typically requires less energy per unit of drilling than RC drilling, its penetration rate is approximately 20% slower. As a result, the longer operational time can lead to higher total energy consumption [10, 11]. Another major limitation is the absence of a feedback loop between diamond core drilling and subsurface conditions. Operators and geologists do not receive real-time information on changes in rock type, structure, or mineralization during drilling, requiring decisions to be made without complete information until the core is retrieved and logged. This limitation becomes increasingly critical as exploration extends to greater depths and, in some settings, targets more complex ore systems, where geological variability can make subsurface characterization more challenging.

**Fig. 1:**
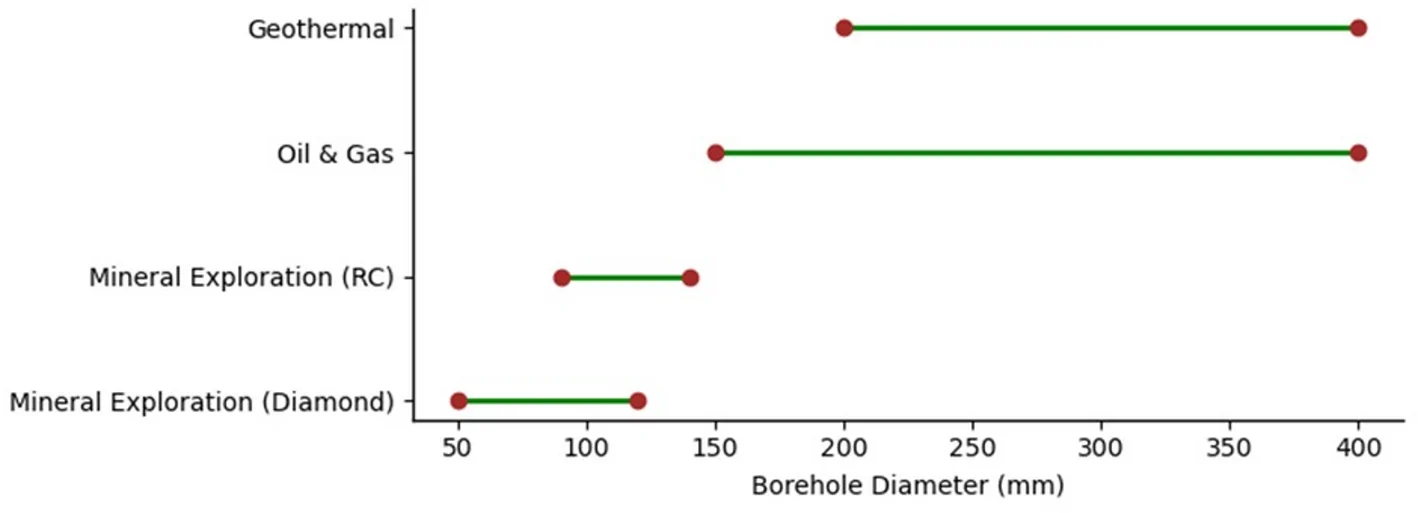
Comparison of typical borehole diameter ranges across different drilling applications [16–18]

**Fig. 2:**
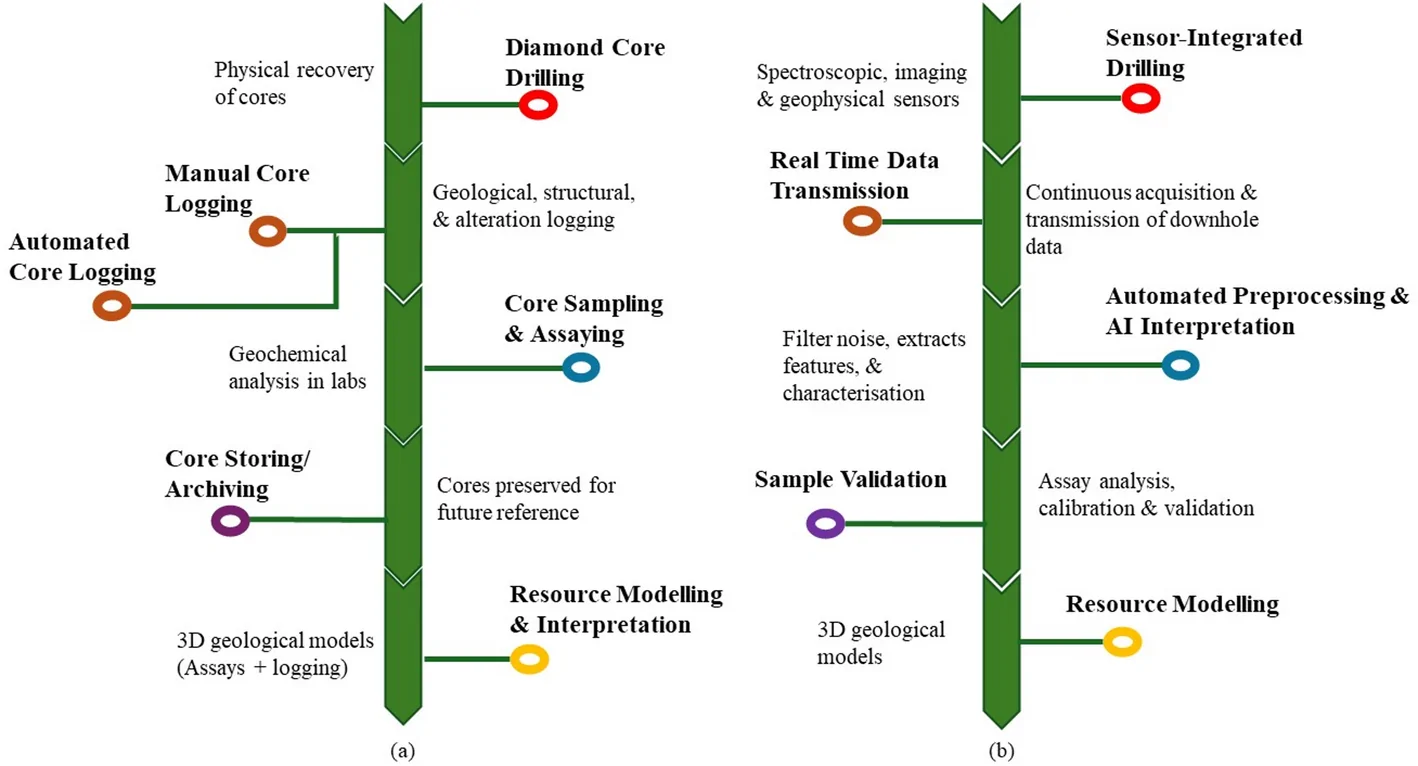
**a** Conventional exploration drilling sequence that depends primarily on diamond core drilling, logging, sampling and assaying versus **b** the proposed sequence involves sensor integrated drilling, which allows for real-time data acquisition, transmission, and automated preprocessing and interpretation using machine learning

These challenges have driven the development of technologies that enhance the acquisition of geological data from drill cores. Recent advancements in automated core scanning technologies have significantly improved both the speed and consistency of data collection. For example, companies such as GEOTEK developed automated multi-sensor platforms that integrate tools like Portable X-ray Fluorescence (pXRF), near-infrared (NIR) spectroscopy, high-resolution cameras, and goniometers to produce detailed mineralogical, geochemical, and structural information [12]. However, these technologies still rely on physical core samples, making them post-coring solutions. Their primary benefit has been the reduction of logging and assaying timelines rather than a decreased dependence on coring itself. Nonetheless, these advancements highlight the increasing potential of sensor-based workflows and serve as a stepping stone toward downhole sensing, which could directly characterize the subsurface during drilling. Early commercial systems show that integrating sensing technology into drilling is technically feasible. For instance, Novamera’s Surgical Mining platform uses a downhole imaging tool to guide precision drilling in real time [13]. The adoption of such technologies in mineral exploration has been slow compared with the oil and gas sector, primarily due to the more challenging downhole conditions. These conditions involve excessive vibrations, high temperatures, abrasive cuttings, and varying drilling fluids, all of which create a tough environment for sensitive equipment. Additionally, mineral exploration boreholes are considerably narrower, typically between 50 and 120 mm diameter compared to those used in geothermal or oil and gas drilling that are 150–400 mm diameter (Fig. 1). This limited space for downhole tools further complicates the integration of instruments. Cost considerations also impact this adoption; oil and gas projects drill to great depths of more than 8 km [14] and generate revenue that justifies investment in advanced downhole technologies, whereas mineral exploration drilling is shallower often extending to around 1000 m [1], making the economic incentive to replace coring less convincing. Efforts such as the coiled-tubing drilling platform, developed through the Deep Exploration Technology CRC and MinEx CRC, illustrate attempts to develop alternative, non-coring drilling approaches for mineral exploration [15]. These technical, environmental, and financial constraints collectively explain the slow uptake of advanced downhole sensors in mineral exploration, despite their proven potential in other industries. Figure 2 illustrates the contrast between conventional workflows and the proposed workflows.

This review explores different technologies for subsurface characterization, assessing their potential to provide geological information comparable to traditional core logging. It focuses on technologies with demonstrated or potential applicability to downhole sensing in mineral exploration, drawing from published literature across the mining, geophysics, petroleum, and geotechnical sectors. Each technology was evaluated based on the geological information it provides, its deployment characteristics, and its technology readiness level (TRL), with TRL assessments derived from the literature. For wireline applications, TRL was primarily based on demonstrated field deployment, commercial availability, and operational maturity in borehole environments. For real-time deployment during drilling, assessments also considered system integration requirements and the sensing mechanism's ability to operate effectively under drilling conditions, including vibration, elevated temperatures, high pressures, and drilling fluids. The review also assesses the potential benefits of these technologies for the mining industry, particularly in enhancing mineral exploration efficiency through real-time subsurface characterization.

## Assessment of emerging downhole sensing, imaging and geophysical technologies for subsurface characterization

### X-ray fluorescence (XRF)

X-ray Fluorescence (XRF) is a widely used analytical technique that provides rapid elemental information to support geological interpretation and geochemical characterization. It can be applied in geological samples, as well as in solids, liquids, and powders by measuring the wavelengths and intensities of X-rays emitted by a sample following excitation [19]. It can analyse (in theory) elements ranging from atomic number 4 (boron) to 92 (uranium) at concentrations from 100% down to a few parts per million (ppm). In mineral exploration, XRF is widely used at surface and in laboratories for rapid geochemical screening of drill cores and chips. Recent studies have shown that XRF technology has the potential to move beyond laboratory and portable applications into real-time ore characterization. Kowalska et al. [20] demonstrated that portable XRF can rapidly and cost-effectively reconstruct downhole lithological profiles by analysing the chemical composition of drill cuttings and core samples. Similarly, Simandl et al. [21] demonstrated the use of portable XRF in exploring carbonatite-hosted niobium deposits. Their study, which included direct measurements on drill core and manual core scanning, revealed strong correlations between pXRF and conventional laboratory analyses for niobium and several other elements, underscoring its potential for making rapid exploration decisions. These studies highlight the versatility of XRF technology in mineral exploration and mining workflows. However, its deployment in downhole environments remains limited, with only a small number of prototype and experimental systems reported [22]. Consequently, it is assigned a TRL of 5–6 for post-drilling (wireline) applications.

XRF presents several advantages for rapid elemental characterization, particularly in core-scanning and drill-site applications. It allows for rapid, non-destructive multi-element analysis with minimal sample preparation, making it ideal for in situ applications where traditional sampling methods are impractical. Its capability to provide direct geochemical measurements, as opposed to relying on indirect proxies, sets it apart from many geophysical techniques and significantly enhances its value for real-time decision-making during drilling. However, integrating XRF into downhole environments poses several challenges. Measurement accuracy is heavily influenced by surface conditions, necessitating consistent contact with the borehole wall and, ideally, a clean measurement surface. XRF may be more suitable for dry borehole conditions, whereas in fluid-filled or rough boreholes, signal attenuation and contamination can compromise data quality [23, 24]. These challenges limit the current readiness of XRF for real-time deployment during drilling, supporting its classification as a TRL 4–5 technology for such applications.

To facilitate effective deployment in real-time downhole sensing, further advancements are needed in tool design, surface interaction mechanisms, and data correction techniques to accommodate variable borehole conditions. With these developments, XRF has the potential to evolve from core-based analysis into a crucial element of real-time geochemical characterization in mineral exploration.

### Laser-Induced breakdown spectroscopy (LIBS)

Laser-induced breakdown spectroscopy (LIBS) is another spectroscopic sensor with potential for downhole use. It is an analytical technique designed to determine the elemental composition of solids, liquids, and gases. The technique utilizes highly energetic laser pulses to vaporize the sample, creating a plasma of ionized matter. The light emitted from this plasma produces spectral signatures that reflect the material's composition [25]. Recent research highlights the strong potential of LIBS in mineral exploration, allowing for the rapid acquisition of compositional data with minimal sample preparation. McMillan et al. [26] demonstrated that capturing the full broadband LIBS emission spectrum between 200–900 nm can effectively identify and distinguish various minerals. LIBS is also particularly effective for the quantitative analysis and stratigraphic correlation of fine-grained rocks, such as basalt, felsite, obsidian, micritic limestone, shale, slate, and marble. It has also proven useful for detecting light-element minerals, including spodumene, chrysoberyl, and homilite, as well as for characterisation of gold-bearing deposits. For instance, Sweetapple & Tassios [27], have demonstrated LIBS's ability to discriminate between spodumene, lepidolite, quartz, and alteration phases in lithium-rich pegmatites. These studies position LIBS as a versatile tool for mineral identification, classification, mapping, and stratigraphic discrimination across diverse geological settings. However, its potential has only been demonstrated in field based and in situ applications [28, 29]. Recent field trials of a prototype downhole LIBS tool, developed by MinEx CRC, have successfully generated multi-element geochemical data during wireline deployment in drill holes, with recent trials demonstrating improved field performance and near real time data acquisition [30]. Consequently, it is classified with a TRL of 4–5 for post drilling (wireline) applications.

LIBS presents several advantages that make it suitable for downhole applications. It allows for real time elemental analysis with high sensitivity and can generate large datasets from each laser pulse [29]. Furthermore, LIBS can identify a wide array of elements, including light elements that are challenging to measure with techniques like XRF. It offers a significant advantage in downhole environments where access and visibility are limited, as it requires minimal sample preparation and can operate effectively without extensive borehole wall cleaning [31]. However, successful implementation will necessitate overcoming challenges such as a clear optical path, low spatial resolution regarding sampling frequency and surface area detection, factors like matrix effects, and plasma variability complicate the accurate conversion of emission intensities into elemental concentrations [32], supporting its current assessment as a TRL 2–3 technology for real-time mineral exploration applications. With advancements in laser miniaturization, optical system design and signal processing, LIBS has the potential to transition from controlled environments to more robust downhole applications.

### Gamma ray spectrometry (GRS)

Gamma ray spectrometry (GRS) was one of the earliest-developed downhole sensing technologies, designed to provide element-specific information from the subsurface. Developed in the 1930s for uranium exploration, the technique was later refined by the oil and gas industry and now has a broader application in geological and environmental sciences. GRS operates by measuring high-energy photons gamma rays emitted during the decay of radioactive nuclei [33]. It is a crucial tool in geophysical logging and plays an essential role in mineral exploration. It can help identify potassic alteration halos, estimate clay content [34, 35], and detect uranium enrichment [36]. For instance, a study by Maden and Akaryalı [37] utilized GRS to map and delineate hydrothermal alteration zones linked to low-sulfidation epithermal gold mineralization in the Eastern Pontides of NE Türkiye. GRS facilitated the identification of variations in radioelement concentrations (K, eU, eTh) within the host rocks, providing a geophysical framework for recognizing alteration patterns that are challenging to detect through surface observations alone. Its extensive application in the mining and petroleum industries indicates a high TRL (8–9) for post drilling applications [35, 38]. Additionally, GRS has been effectively integrated into logging while drilling systems in the oil and gas sectors [39], showcasing a high TRL (7–8) for real time deployment. This high level of technological maturity is due to several inherent advantages of GRS. These includes its capability to simultaneously identify and quantify multiple radionuclides, minimal sample preparation, rapid data acquisition, and the generation of continuous downhole profiles. Additionally, it can sense beyond the borehole wall, providing a more representative measurement of the surrounding formation [40]. Despite its advantages, GRS has significant limitations. It offers limited lithological resolution since the abundances of radioelements do not uniquely indicate mineralogy [41]. Furthermore, GRS is not effective if the target minerals do not contain sufficient radioactive elements. Additionally, conventional analyses often overlook portions of the spectrum associated with other elements [38].

Recent advancements in detector sensitivity, spectral resolution, data processing, and digital integration have greatly improved the capabilities of GRS for real-time downhole sensing in mineral exploration [42]. These innovations enhance GRS's applicability in mineral exploration drilling, allowing rugged, miniaturized detectors to function in high-temperature and high-pressure environments without needing a clear optical path. Although GRS has limitations for non-radiogenic ores, it can still provide valuable complementary data on lithology and alteration. When combined with other geophysical or spectroscopic tools, GRS is one of the most promising technologies for near-term deployment and integration into next generation downhole sensing frameworks.

### Neutron-based geophysical techniques (NbGT)

Neutron-based geophysical techniques (NbGT) offer an alternative method for elemental analysis by exploiting neutron interactions with formation materials, allowing for in situ characterization of elemental composition in downhole environments. These techniques generally involve emitting high-energy neutrons into the surrounding formation, followed by detecting the resulting gamma rays or scattered neutrons, which can be correlated to elemental composition [43]. Common methods include natural gamma, gamma–gamma, and prompt gamma neutron activation analysis (PGNAA), all of which are widely used for in situ formation evaluation [44]. Neutron activation methods are especially effective for detecting elements that are challenging to measure with conventional spectroscopic techniques and have been widely utilized in borehole geophysics for petroleum exploration. Their success has also led to their adoption in uranium and coal mining industries, where they help estimate parameters such as ash content, lithology, and elemental composition [45]. For instance, PGNAA has been effectively applied in coal logging to assess ash and iron content, as well as seam thickness in both dry and water-filled boreholes [45]. Similarly, field trials of PGNAA-based systems, like spectrometric logging method and instrumentation (SIROLOG), have shown reliable estimation of copper grades in porphyry deposits, capturing downhole grade variability [46].

The established application of these techniques across the oil and gas, coal, and uranium sectors [45, 46] reflects a high level of technological maturity (TRL 8–9) for downhole applications, particularly in wireline logging environments. For instance, recent industry systems, such as Epiroc's OreSight, have showcased the potential of pulsed neutron tools to provide high-resolution, large-volume assay data directly from drill holes [47]. However, their use in mineral exploration and real-time while-drilling environments is still limited. Performance is highly sensitive to formation composition, borehole fluids, and environmental conditions, which can complicate interpretation and reduce accuracy. Additionally, neutron activation methods are often element-specific, with their effectiveness depending on the target element, matrix composition, and detector configuration. Sensitivity can also be reduced at low element concentrations, restricting their applicability in certain exploration scenarios [43, 48]. These limitations suggest a moderate level of technological maturity for real-time deployment (TRL 4–5), despite their high maturity in post-drilling applications.

To advance NbGT for real-time deployment, targeted developments in tool design and system integration are essential. This includes miniaturizing neutron sources and detectors for incorporation within the bottom hole assembly, enhancing shielding and safety systems for operation during drilling as nuclear tools involve radioactive emitting sources, and developing robust calibration to address borehole and environmental effects. Additionally, faster data acquisition and processing workflows will be necessary to facilitate near real-time interpretation.

### Hyperspectral imaging (HSI)

High-resolution spectral imaging techniques allow for the identification of mineralogical variations through their distinct spectral signatures, with hyperspectral imaging (HSI) being one of the most used methods. It is a technique that originated from remote sensing and earth monitoring. Developed by NASA's jet propulsion laboratory in the 1970s, HSI was designed to study the Earth's surface. The technique enables the identification of objects by analysing their interactions with electromagnetic radiation, as each object possesses a unique spectral signature characterized by the specific wavelengths of light it absorbs and reflects [49]. HSI has been successfully utilized to characterize and map a variety of minerals and alteration assemblages that can be used to provide lithological interpretation, including hydrothermal alteration systems such as epithermal and porphyry deposits, as well as iron-bearing minerals, carbonates, and clays [50]. One of the key advancements in utilizing HSI for mineral exploration is core scanning. This technique allows for high-resolution, non-destructive imaging, enabling rapid mineralogical characterization of drill cores, with the minerals identified depending on the spectral range of the sensor, such as short-wave infrared (SWIR) and thermal infrared (TIR) [51]. As a result, it enhances the speed of geological interpretation and supports more informed decision-making in exploration workflows [52].

As a potential downhole tool, HSI could offer valuable insights into mineralogy. However, adapting HSI for downhole applications presents several technical challenges as it requires controlled lighting and a clear optical path to the borehole wall conditions that are significantly hindered by drilling fluids, cuttings, and mud which can degrade both spatial and spectral resolution. Furthermore, the physical constraints of the bottom hole assembly (BHA) restrict sensor size, positioning, and stability, making it challenging to maintain consistent imaging during drilling and affect data quality. The high-pressure, high-temperature, and abrasive environments further complicate these issues by degrading electronics and inducing mechanical stress on the components. Additionally, HSI mainly captures the mineralogy of the borehole wall rather than providing volumetric information about the surrounding rock mass, which requires integration with other sensing technologies to gain a more comprehensive understanding of the subsurface. Its effectiveness is also limited by its spectral range, as different wavelength regions are sensitive to specific mineral groups [51]. As a result, only minerals with distinctive spectral features within the chosen spectral range can be accurately identified [53]. While HSI is a powerful and mature tool for surface-based exploration and core scanning [49, 54], its direct application as a real-time downhole sensor is limited, corresponding to a low TRL of 2–3 for real time deployment and wireline applications. It should be viewed as a long-term experimental option rather than a near-term solution.

### Light detection and ranging (LiDAR)

Light detection and ranging (LiDAR) provide a unique advantage as an imaging tool by providing 3D reconstructions of borehole geometry. It operates on the principle of light reflection, using a laser to emit pulses toward an object and measuring the time it takes for the pulse to return to the sensor. LiDAR systems are highly effective at generating high-resolution 3D models and images, offering detailed insights into the shape and texture of exposed surfaces [55]. These capabilities make LiDAR a valuable tool in mineral exploration, especially for mapping geological structures, identifying surface features, and assisting in target generation. In recent years, the mining industry has increasingly adopted LiDAR technology to enhance safety, mapping accuracy, and operational efficiency. By capturing detailed geometries of discontinuities, LiDAR enables the derivation of essential rock mass parameters, such as rock quality designation (RQD), fracture spacing, and orientation of discontinuities, thereby supporting more reliable geotechnical assessments [56–58].

The use of LiDAR in borehole environments has been limited because it requires a clear optical path and an unobstructed line of sight. Maintaining these conditions in downhole environments is challenging due to the presence of drilling fluids, cuttings, and uneven borehole walls. These factors scatter and attenuate laser signals, reducing both measurement accuracy and resolution [55]. Moreover, the narrow diameter of mineral exploration boreholes restricts the integration of necessary scanning mechanisms for effective spatial coverage. Achieving consistent orientation and full 360° scanning in these confined spaces adds further complexity to deployment. Additionally, LiDAR cannot penetrate the borehole wall, which means it cannot directly detect structural features like faults, folds, or fractures [59]. It is therefore assigned a TRL of 3–4 for post drilling applications and a low TRL of 2–3 for real-time deployment. Despite these challenges, LiDAR has potential for post drilling applications in dry or controlled borehole environments, where it can deliver high resolution structural information. However, its use for real time deployment is still limited, requiring significant advancements in system design and environmental tolerance to ensure reliable operation. At the same time integrating LiDAR with machine learning offers opportunities to enhance data interpretation and decision making [60, 61].

### Optical televiewer (OTV)

Downhole structural characterization primarily depends on imaging tools like the optical televiewer (OTV), which offers high-resolution, continuous images of boreholes using a modified wide-angle lens that enables 360° surveying [62]. These images are oriented to magnetic north and can be presented as unwrapped cylinders with a horizontal scale or wrapped back into cylindrical form to create a virtual core, aiding in core orientation and addressing issues of core loss or poor recovery [63]. The images clearly display all beddings, joints, and fractures, including their inclinations and orientations. According to Williams & Johnson [64] OTV is extensively used in geotechnical, hydrogeological, oil and gas industry for high-resolution, oriented imaging of borehole walls. Its primary applications include structural analysis, reservoir characterization, fracture identification, and well quality assurance, indicating a high TRL of 8–9 for post drilling deployment [63–65]. However, its real time application is hindered by the need for a clear optical path and stable borehole conditions. Active drilling environments are often characterized by turbid drilling fluids and unstable borehole walls, which degrade image quality and limit visibility. Additionally, the instrument is sensitive to vibrations and requires precise centering [62]. Consequently, it has a low TRL of 2–3 for real time deployment. while it provides valuable geological and structural information, its current design restricts real-time deployment. Significant advancements in borehole conditioning, optical robustness, and real-time image processing are essential for integrating these technologies into real-time deployment systems.

### Acoustic televiewer (ATV)

Unlike the OTV the acoustic televiewer (ATV) uses acoustic signals rather than light to generate images of the borehole wall. It creates an image of a borehole wall by utilizing the travel time and amplitude of an acoustic signal transmitted and received by a rotating ultrasonic mirror sensor [66]. This tool scans the borehole walls, allowing for the identification of fractures, fissures, veins, bedding planes, and changes in lithology based on the variance in the acoustic properties of the formation [65]. This system is utilized in mining, oil and gas, and hydrogeology as a post-drilling imaging tool [67], producing high resolution acoustic images that show open fractures and fracture density, which is critical for identifying permeable and high hydro conductivity in geothermal systems hence a high TRL (8–9) for post drilling (wireline) deployment. Kao et al. [68] conducted a study demonstrating the application of ATV technology in geotechnical techniques. The research demonstrates that, in addition to visualizing borehole structures, ATV data can reliably correlate acoustic amplitude with essential geotechnical parameters. The authors also introduce a new rock mass evaluation index, Aco-RQD, which broadens the applicability of ATV logging in geotechnical engineering practices.

ATV operates effectively in opaque or muddy borehole conditions by utilizing acoustic waves, which eliminates the requirement for a clear optical path. This feature allows the ATV to adapt to a broader range of drilling environments including mud filled or turbid boreholes where optical imaging systems are ineffective, suggesting potential for integration into active drilling workflows and thereby placing the system at TRL 5–6. However, its performance remains dependent on the presence of a fluid-filled borehole to transmit ultrasonic pulses between the tool and the borehole wall. In dry or wet drilled holes, which are common in hard-rock exploration, this acoustic coupling is disrupted. As a result, signal attenuation occurs, hindering reliable image acquisition. While fluid conductivity and clarity typically do not impact signal transmission, thick drilling muds and mudcake buildup can compromise image quality [69]. Additionally, the presence of magnetic minerals and steel casing can distort acoustic signals, reducing the accuracy of the resulting images [67]. To improve the effectiveness of ATV systems for mineral exploration, further advancements in tool miniaturization, real-time data processing, resolution, and adaptability to different drilling fluids are essential. These developments are crucial for transforming ATV from primarily a post drilling tool into a dependable tool for real-time drilling sensing system.

### Borehole transient electromagnetic method (BTEM)

The borehole transient electromagnetic method (BTEM) is a highly effective geophysical technique for mineral exploration [70]. This technology utilizes either an ungrounded loop source or a ground line source to emit a single pulsed magnetic field into the ground. It then monitors the changes in the secondary electromagnetic field resulting from the underground medium's response, allowing for an in-depth analysis of the formation's electrical structure [71]. It has been deployed to depths of approximately 2000 m, with reported detection ranges of 200–300 m around the borehole[72]. BTEM is particularly adept at identifying sulphide and other conductive ore bodies, which exhibit distinct electromagnetic responses, making it valuable for the exploration of conductive mineralisation such as magmatic Ni-Cu sulphide and volcanogenic massive sulphide (VMS) deposits [73]. The method offers enhanced resolution and penetration compared to surface electromagnetic techniques, as the receiver is positioned within the drill hole and closer to potential targets. This configuration minimizes signal attenuation and increases sensitivity, enabling more precise mapping of geological structures, depth profiling of ore bodies, and delineation of conductive zones that may not be directly accessed through drilling [72].

Zhi et al. [74] utilized BTEM configuration at the Wulong Gold Deposit, incorporating a 3004-m exploration borehole. By deploying downhole receivers along the borehole, they facilitated rapid anomaly localization through an eddy-current loop approach. The method effectively delineated anomalies at depths exceeding 2,800 m, showcasing superior resolution compared to traditional surface TEM. This capability indicates a high TRL of 7–8 for post-drilling (wireline) deployment in relevant environments. However, several challenges constrain the integration of BTEM into real-time downhole sensing systems resulting in a TRL of 4–5. For example, its sensitivity decreases significantly for weakly conductive or non-conductive minerals like quartz, feldspar, and oxides, which limits its applicability to specific types of ore deposits [75]. Additionally, interpreting BTEM data can be challenging, particularly in geologically complex areas. Overlapping conductors and nearby infrastructure, such as power lines and pipelines, can introduce geological noise and create false anomalies, increasing the risk of misinterpretation [76]. Modern innovations in BTEM technology have concentrated on enhancing hardware, refining data processing, and integrating modern analytics. These improvements have led to greater sensitivity, deeper penetration, and more efficient surveys [77]. Furthermore, integrating BTEM data with machine learning and AI-driven analytics accelerates anomaly detection and strengthens correlations with geological and geochemical datasets, resulting in higher exploration success rates [78]. BTEM is a promising emerging technology for real-time downhole sensing. Although it is currently limited to post-drilling wireline deployment, further development could allow for drilling-integrated or near-real-time applications.

### Borehole ground penetrating radar (GPR)

This technique uses the relationship between wave velocity and material properties to explore the shallow subsurface of the earth. Ground Penetrating Radar (GPR) emits electromagnetic (EM) waves as microwave pulses with frequencies ranging from 10 MHz to 1.5 GHz [79]. Unlike many other geophysical tools, GPR can detect both metallic and non-metallic objects and is effective for mapping subtle differences in density and porosity. Compared to LiDAR, GPR can penetrate rock surfaces and detect fractures, voids, or lithological boundaries [80]. It is less sensitive to muddy or debris-filled conditions, making it suitable for downhole applications. However, GPR has limitations; conductive formations such as shales, graphitic rocks, and sulphides attenuate the signals, which restricts penetration [81]. Additionally, it offers lower resolution compared to LiDAR and Hyperspectral Imaging (HSI). Recent innovations in GPR have significantly enhanced resolution and depth accuracy [82]. Traditionally, GPR systems relied on manual interpretation, but the integration of artificial intelligence has automated this process, leading to faster and more precise results. For instance, P. Liu et al. [83] applied deep learning models for tunnel inspections, which effectively addressed issues of human error and inefficiency in interpreting GPR data. Their approach automatically detected structural defects, demonstrating the potential of deep learning to improve the efficiency, accuracy, and practicality of GPR-based assessments. Additionally, the development of lightweight, flexible, and portable GPR systems is broadening their applicability in different fields. It has been successfully deployed in mineral exploration, geotechnical, tunnelling, and oil and gas applications, demonstrating its effectiveness for subsurface imaging in borehole environments [81, 84]. This corresponds to a high TRL 7–8 for post drilling (wireline) deployment. However, its application for real-time downhole sensing remains limited, as most reported applications are confined to post-drilling or surface-based surveys [80, 85], resulting in a TRL of 4–5.

The performance of GPR is strongly influenced by significant signal attenuation in conductive host rocks, which restricts its depth of penetration and data quality. To overcome its limitations in conductive settings, it can be integrated with BTEM methods, which are particularly sensitive to conductive ore bodies such as massive sulphides. Together, these techniques can bridge the gap between structural context and ore targeting, providing a more integrated downhole exploration approach, with GPR mapping structures in resistive rocks and BTEM detecting conductive mineralization, ensuring broader geological coverage. Integrating GPR and BTEM could, in theory, offer both structural and mineralization information from a single downhole platform. However, practical implementation would need to address challenges such as electromagnetic interference between the systems, the need for tool miniaturization to fit narrow drillholes which are typically 50–120 mm in diameter [16], and the requirement for reliable real-time data transmission in drilling conditions. This necessitates the development of compact, robust multi-sensor systems that can maintain signal integrity while delivering sufficient signal strength and resolution.

### Muon tomography

Muon tomography is a passive geophysical imaging technique that measures the attenuation of natural cosmic-ray muon flux as it traverses matter. By detecting variations in muon intensity with specialized sensors, 2D and 3D images of subsurface density distributions can be constructed [86]. Muography has emerged as a powerful tool for mineral exploration and subsurface monitoring [87], offering several advantages over traditional geophysical methods. Since its inception as a resource exploration technique in the 1970s, muography has undergone significant advancements. Recent innovations, such as the Geoptic borehole muon imaging system [88], highlight its evolution towards post-drill applications. This system features ruggedized detectors, approximately 60 mm in diameter and 2.2 m in length, engineered to withstand harsh downhole conditions, including high humidity and underground water. This marks a significant step toward utilizing muography for subsurface characterization and potentially for downhole sensing during drilling.

Numerous studies have confirmed muography's ability to reconstruct mineral deposits of varying geometries [89, 90]. By providing information on the size and spatial extent of mineralized bodies, muography can aid in characterizing exploration targets and enhance understanding of their three-dimensional structure. Its key advantages include being a passive technique that requires no active source and its high penetration depth, which allows for imaging through hundreds of meters of rock. Additionally, muography is largely insensitive to electromagnetic and mechanical noise, making it particularly suitable for complex geological environments, where heterogeneous structures and significant density contrasts can limit the effectiveness of conventional geophysical methods [86]. However, the technique has notable limitations. Its effectiveness decreases in low-density-contrast ore bodies, and uncertainties associated with muon scattering and signal attenuation can complicate interpretation. Additionally, the relatively long acquisition times and limited spatial resolution restrict its application to detailed characterization of selected targets rather than broad exploration screening.[91]. Muography shows promise for subsurface density imaging, and recent advancements in borehole detector design have demonstrated that ruggedized sensors can be deployed downhole [88]. The technique currently has a TRL of 6–7 for wireline applications [87, 89], but remains at an earlier stage for real-time deployment during drilling, with a TRL of 2–3. Continued improvements in detector sensitivity, data acquisition rates, and automated noise discrimination could improve its suitability for real-time downhole sensing.

### Seismic while drilling (SWD)

The concept of using the drill bit as a seismic source originated in the late 1980s [92]. Seismic While Drilling (SWD) is a geophysical technology that utilizes the seismic waves generated by the drill bit or a dedicated downhole source to image the subsurface. Receivers placed on the surface or in adjacent boreholes capture these waves and produce digital seismograms [93]. SWD provides numerous advantages, including the ability to deliver structural information about surrounding rocks, identify faults and geological anomalies, support basin and reservoir modelling, and predict pore pressure ahead of the drill bit [92, 94]. It has proven effective in the oil, gas, and geothermal industries for real-time stratigraphy mapping and hazard detection ahead of the drill bit. For instance, Kim et al. [95] applied SWD along with borehole televiewer data on Seokmo Island, South Korea, successfully identifying fracture locations, determining their orientations, and constructing an enhanced subsurface structural model. Their research demonstrated that SWD based methods can reliably image fracture systems and improve structural interpretation as such, it demonstrates a high TRL of 7–8 for real-time deployment. However, its transferability in mineral exploration remains limited due to several factors. Oil and gas operations focus on delineating large stratigraphic and structural traps, while mining exploration prioritizes high-resolution mineralogical and structural information, a level of detail that seismic methods often fail to achieve [96]. Additionally, the quality of seismic data in hard rock environments is compromised by weak drill-bit sources and high background noise, which limits the clarity and interpretability of the results [97]. Furthermore, the smaller borehole diameters typical in mineral exploration further restrict sensor deployment and reduce signal strength.

If ongoing efforts to miniaturize tools and develop higher-frequency downhole seismic sources succeed, SWD could transform mineral exploration. These advancements are expected to significantly improve resolution and sensitivity, allowing for the detection of smaller structural features such as fractures, faults, and cavities, which are essential for accurately delineating ore bodies. At the same time, researchers are increasingly investigating the potential of drill-bit-generated vibrations as alternative seismic source. Accelerometer-based measurements of these vibrations, analysed using machine learning techniques, have shown promise for real-time lithological identification [98–100]. Together, these developments indicate a future where SWD, combined with intelligent vibration analysis, can provide continuous geotechnical and geological information throughout the drilling process.

## Discussion

**Fig. 3:**
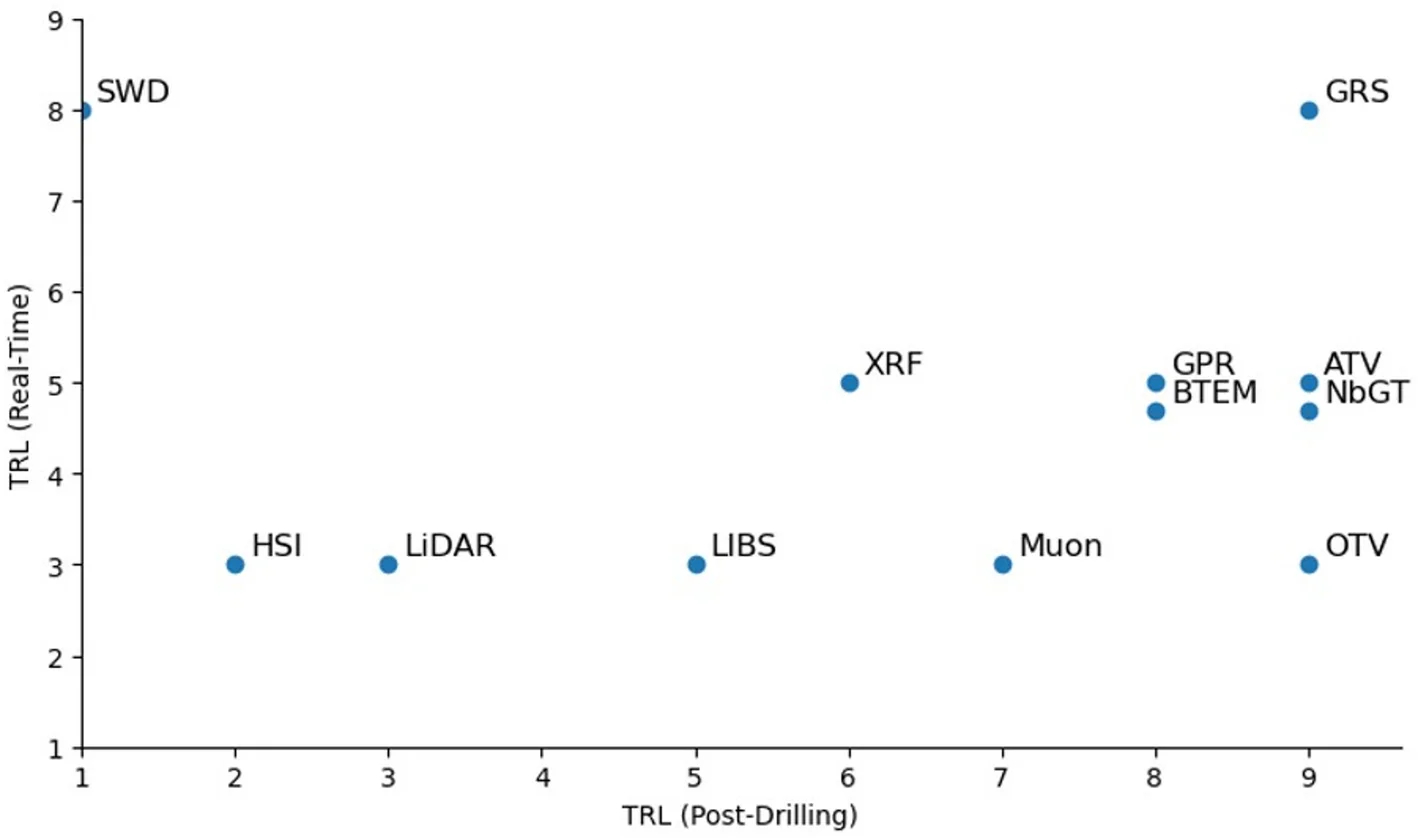
Assessment of technological readiness for downhole sensing technologies, applicable to both post-drilling (wireline) and real-time (while-drilling) deployment

As summarized in Table 1 and Fig. 3, the technological readiness of downhole sensing technologies differs significantly between post-drilling (wireline) and real-time (while-drilling) applications. Although several sensing techniques are highly mature for post-drilling use, their readiness for real-time integration is often limited by operational and environmental challenges encountered during active drilling. Technologies that are well-established in harsh borehole environments, particularly those commonly used in the oil and gas industry, such as GRS and SWD, demonstrate high readiness for both post-drilling and real-time applications. In contrast, electromagnetic methods like GPR, and BTEM systems perform well in post-drilling scenarios but demonstrate moderate readiness for real-time use. This is mainly because they are sensitive to formation properties and operational constraints, such as signal attenuation in conductive environments, interference from the drill string, and the need for stable sensor positioning and controlled acquisition conditions. Optical and imaging-based techniques, including HSI, LiDAR, and OTV, are largely confined to post-drilling applications. Their dependence on clear optical paths, controlled illumination, and stable borehole conditions significantly limits their feasibility for real-time deployment in dynamic drilling environments. The findings indicate that although many technologies are well established for post-drilling applications, their readiness for real-time deployment is still limited.

**Table 1:** Summary of sensor readiness for downhole integration

Sensor Technology	Principle of Operation	Information Captured/ Strength	Spatial information/ dimensionality	Application	Limitations	TRL (Wireline)	TRL (Real-time)
X-ray Fluorescence (XRF)	Measures the wavelengths and intensities of X-rays emitted by a sample	1.Elemental composition 2. Qualitative, semi-quantitative analysis 3.Minimal sample preparation	1. Point/ continuous elemental measurements along the borehole or core 2. 1D	1. Geochemical characterization 2. Target delineation	1. Shallow penetration 2. Surface irregularities reduce accuracy	5–6 [22, 101, 102]	4–5 [23, 24]
Laser-Induced Breakdown Spectrometry (LIBS)	Excites atoms with a pulsed laser, then analyses emitted plasma spectra	1.Direct, multielement geochemical data 2. Qualitative, semi-quantitative analysis	1. Point/ continuous measurements along the borehole 2. 1D	1. Geochemical characterization 2. Target delineation	1. Requires a clear optical path 2. Low spatial resolution 3. Matrix effects/ Plasma variability affects the accuracy	4–5 [28–30]	2–3 [32]
Gamma Ray Spectrometry (GRS)	Measures natural gamma emissions	1. Natural radioactivity 2. Lithological boundaries 3. Unaffected by optical clarity	1. Continuous measurements along the borehole 2. 1D	1. Lithological characterization 2. Alteration Mapping 3. Resource definition	1. Low spatial resolution 2. Ineffective for non-radiogenic ores 3. Affected by casing and background noise	8–9 [35, 38]	7–8 [39, 40]
Neutron-Based Geophysical Techniques (NbGT)	Measures gamma rays or scattered neutrons resulting from neutron induced reactions	1.Elemental composition 2.Grade distribution 3.Lithology	1. Continuous measurements along the borehole 2. 1D	1. Lithological characterization 2. Resource characterization	1.Sensitive to formation and borehole conditions 2. Low sensitivity at low concentrations 3.Radiation safety concerns	8–9 [45–47]	4–5 [43, 48]
Hyperspectral Imaging (HSI)	Captures reflectance of electromagnetic waves across many spectral bands	1. Detailed mineralogical composition 2. Alteration mapping	1. Continuous hyperspectral images of the core surface 2. 1D-2D	1. Mineralogical characterization 2. Alteration mapping 3. Lithological characterization	1. Requires illumination 2. Requires clear optical path 3. Limited penetration	2–3 [49, 54]	2–3 [51, 53]
Light Detection and Ranging (LiDAR)	Uses laser pulses to measure the return time of reflected signals	1.Structural orientation 2.Cavity Mapping 3.Borehole geometry 4.High spatial resolution	1. Continuous measurements of borehole wall geometry 2. 2D-3D	1. Structural characterization 2. Geotechnical characterization 3. Borehole geometry	1. Limited penetration 2. Line of sight required 3.Sensitive to moisture, dust	3–4 [59]	2–3 [55]
Optical Televiewer (OTV)	Uses a downhole camera with a rotating mirror to capture continuous 360° images of the borehole wall	1.High-resolution borehole imagery 2.Fracture orientation	1. Images of the borehole wall 2. 2D surface	1. Structural characterization 2. Geotechnical characterization	1. Requires clear borehole fluid 2. Requires a clear optical Path 3. The tool must be centralised 4. Limited penetration	8–9 [63–65]	2–3 [62]
Acoustic Televiewer (ATV)	Emits ultrasonic pulses, records travel time and amplitude of reflections to map borehole wall	1.Borehole wall structure 2.Fracture mapping 3.Operates in opaque fluids	1. Acoustic images of the borehole wall 2. 2D surface	1. Structural characterization 2. Geotechnical characterization	1.Needs fluid coupling 2. Low resolution	8–9 [67, 68]	4–5 [67, 69]
Borehole Transient Electromagnetic method (BTEM)	Measures decay of induced EM fields using a borehole receiver	1.Sulfide zones 2.Electrical conductivity distribution 3.Indirect Mineralogy	1. Continuous electromagnetic measurements along the borehole, interpreted into surrounding formation 2. 2D-3D	1. Conductive mineralization targeting 2. Orebody characterization	1. Conductive material could obscure the signal 2. Nonspecific-graphite or clay might be mistaken for sulfides	7–8 [72, 74]	4–5 [75, 76]
Borehole Ground Penetrating Radar (GPR)	Emits radio/ microwaves and measures reflections	1.Structural orientation 2.Stratigraphy 3. Voids	1. Continuous subsurface reflection measurements along the borehole, interpreted beyond the borehole wall 2. 2D-3D	1. Target delineation 2. Drill guidance 3. Subsurface characterization	1. Attenuated in conductive formations	7–8 [81, 84]	4–5 [80, 85]
Muon Tomography	Measures the attenuation of natural cosmic-ray muon flux as it traverses matter	1. Large volume density mapping 2. Large scale geological structures 3. Passive	1. Path integrated measurements through the subsurface 2. 3D volumetric information	1. Orebody characterization 2. Target delineation	1. Long acquisition times 2. Presence of soft and fake muons affect signal accuracy 3. Coarse resolution	6–7 [87, 89]	2–3 [91]
Seismic while drilling (SWD)	Uses the drill bit, or a downhole seismic source, signals recorded at the surface	1. Rock hardness 2. Formation boundaries 3. Lithological transitions 4. uses existing	1. Continuous seismic measurements during drilling, interpreted into the surrounding formation 2. Potentially 3D	1. Drill guidance 2. Subsurface characterization	1. Weak/ Noisy seismic waves from the drill bit 2. Resolution decreases with depth		7–8 [92, 94, 95]

1D, 2D, and 3D refer to one-dimensional, two-dimensional, and three-dimensional spatial information, respectively. This stated dimensionality represents the primary spatial representation of the recorded or interpreted information, and not the full physical volume investigated by the sensor.

Collectively, these technologies represent significant progress in acquiring lithological, mineralogical, alteration, geochemical, 3D mapping, and structural information directly from boreholes. They offer a means to reduce reliance on traditional diamond coring in mineral exploration. The future of downhole sensing is likely to transition from single-sensor deployment to integrated, multi-sensor systems. For instance, electromagnetic methods like BTEM systems, which are highly sensitive to conductive mineralization, can be combined with GPR, which excels in resistive environments. This combination offers a more comprehensive understanding of subsurface conditions. Likewise, geochemical sensors such as XRF or LIBS can be paired with structural imaging tools like ATV or OTV to simultaneously capture the elemental composition and structural features of the borehole wall. Despite these advantages, integrating multiple sensing technologies presents several challenges. These challenges include mutual electromagnetic interference between active sensing systems, limited space within narrow boreholes, and increased power and data transmission requirements, and differences in data formats, sampling intervals, spatial and temporal resolution, depth references and calibration procedures. These interoperability challenges are particularly important because the value of multi-sensor sensing depends not only on acquiring multiple datasets, but also on accurately aligning, standardizing, and integrating them into a common geological framework.

Another critical challenge in real-time downhole sensing is data telemetry, which involves the reliable transmission of large volumes of data from the bottom of the borehole to the surface. As sensing systems evolve to become more sophisticated, the volume, velocity, and complexity of the generated data significantly increase, placing substantial demands on telemetry infrastructure. In the oil and gas industry, various telemetry methods have been developed to meet these challenges, including mud-pulse telemetry, electromagnetic (EM) telemetry, and wired drill pipe or fibre-optic systems [103]. Mud-pulse telemetry is one of the most common methods for transmitting data, as it sends information through pressure pulses in drilling fluid. However, this method is limited by relatively low data rates and signal attenuation in deep or complex wells [104]. Electromagnetic telemetry provides an alternative that functions without fluid circulation, but it is highly sensitive to formation conductivity and depth [103]. More recently, wired drill pipe and fibre-optic systems have emerged, allowing for high-bandwidth, near real-time data transmission, which significantly enhances data rates and reliability [105, 106]. Furthermore, fibre optic sensors can be deployed through post drilling (wireline) providing flexibility [107]. Despite these advancements, adapting these telemetry systems for mineral exploration drilling presents additional challenges, including smaller borehole diameters, and harsher drilling conditions. These limitations highlight that telemetry continues to be significant bottleneck in real-time downhole sensing systems.

The need for quantitative chemical (assay) data, checked against certified reference materials, to determine deposit grades, as well as detailed spatially resolved mineralogical studies for process mineralogy and geometallurgy are challenges that no sensors deployed so far can satisfy. Drill cuttings returned to the surface can be sampled for analysis but with less spatial precision than in a diamond core, and with risk of fractionation between grain sizes [111].

As integrated downhole sensing systems generate larger and more complex volumes of data, ML will play an essential role in data processing, interpretation and real-time decision making. ML techniques have shown significant promise in mineral exploration especially in handling complex, high dimensional datasets from geophysical, geochemical and spectral measurements. For instance, ML techniques are applied to hyperspectral imaging data for mineral mapping and alteration detection [97, 108]. Similarly, supervised learning methods such as convolutional neural networks (CNN), recurrent neural networks (RNNs), and long short-term memory (LSTM) networks are commonly employed for lithological classification, utilizing features from geophysical logs, geochemical data to automatically categorize rock types and mineralized zones. For example, Komadja et al. [109] utilized ML techniques such as support vector machines, random forests, and extreme gradient boosting (XGBoost) for lithology classification based on MWD data, while Qingfeng et al. [110] proposed a comprehensive machine learning framework for lithology identification during drilling, combining kernel principal component analysis, clustering, and ensemble learning (stacking). This highlights the importance of machine learning in facilitating the integration and real-time analysis of complex, multi-sensor downhole datasets.

**Fig. 4:**
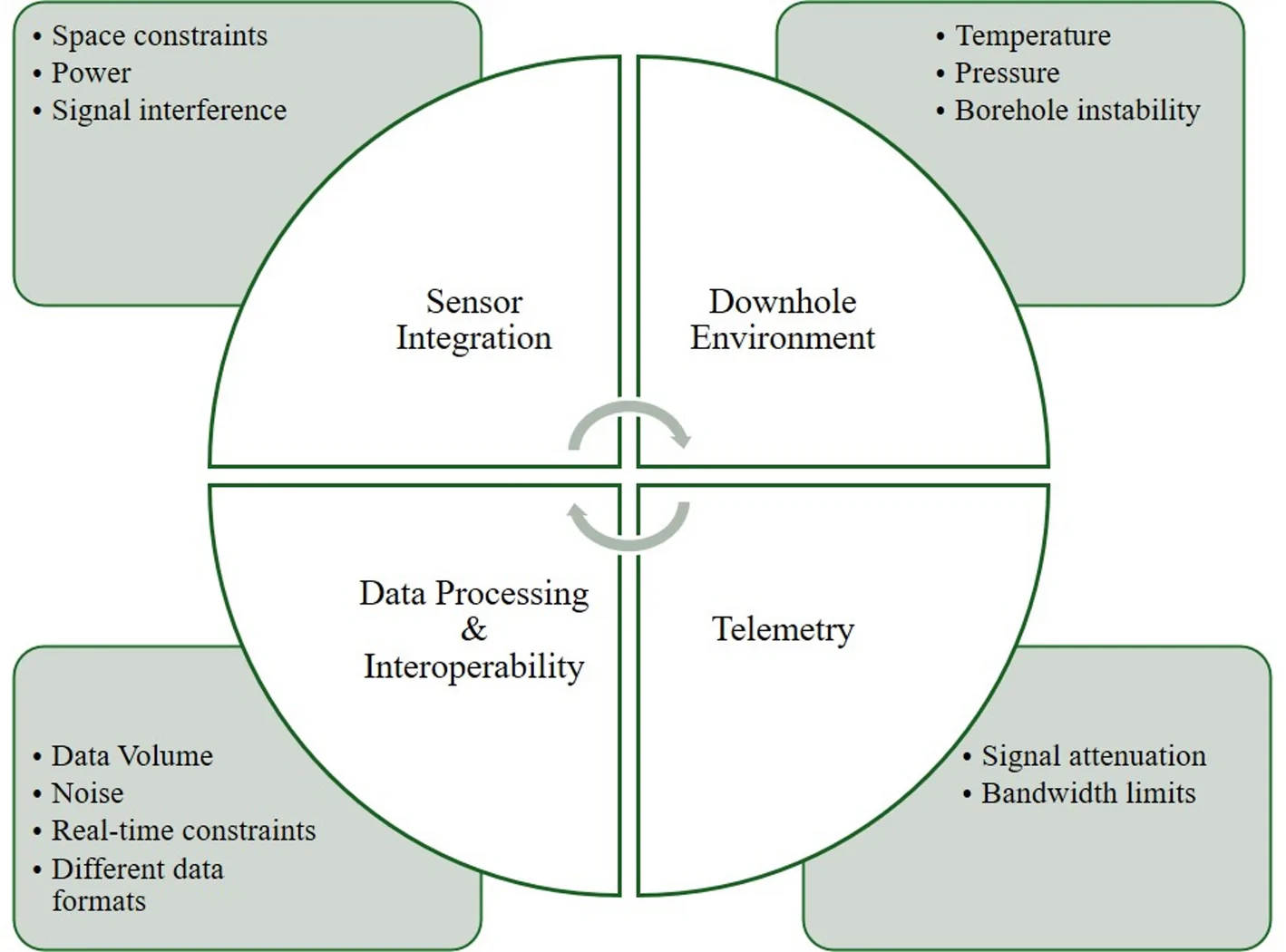
Interconnected challenges in integrated downhole sensing systems include limitations in sensor integration, environmental constraints, telemetry issues, and difficulties in data processing

This review emphasizes that although various sensing technologies are available and show strong performance in post-drilling applications, their implementation in real-time, downhole sensing scenarios is hindered by a combination of environmental, operational, and system constraints. These challenges go beyond the performance of individual sensors and encompass issues related to sensor integration, telemetry bandwidth, and the management of large, multi-modal datasets. The relationship is illustrated in Fig. 4. The findings suggest that the primary obstacle to progress is not the absence of suitable sensing technologies, but the complexities involved in deploying, integrating, and validating these systems within the constraints of downhole environments.

**Fig. 5:**
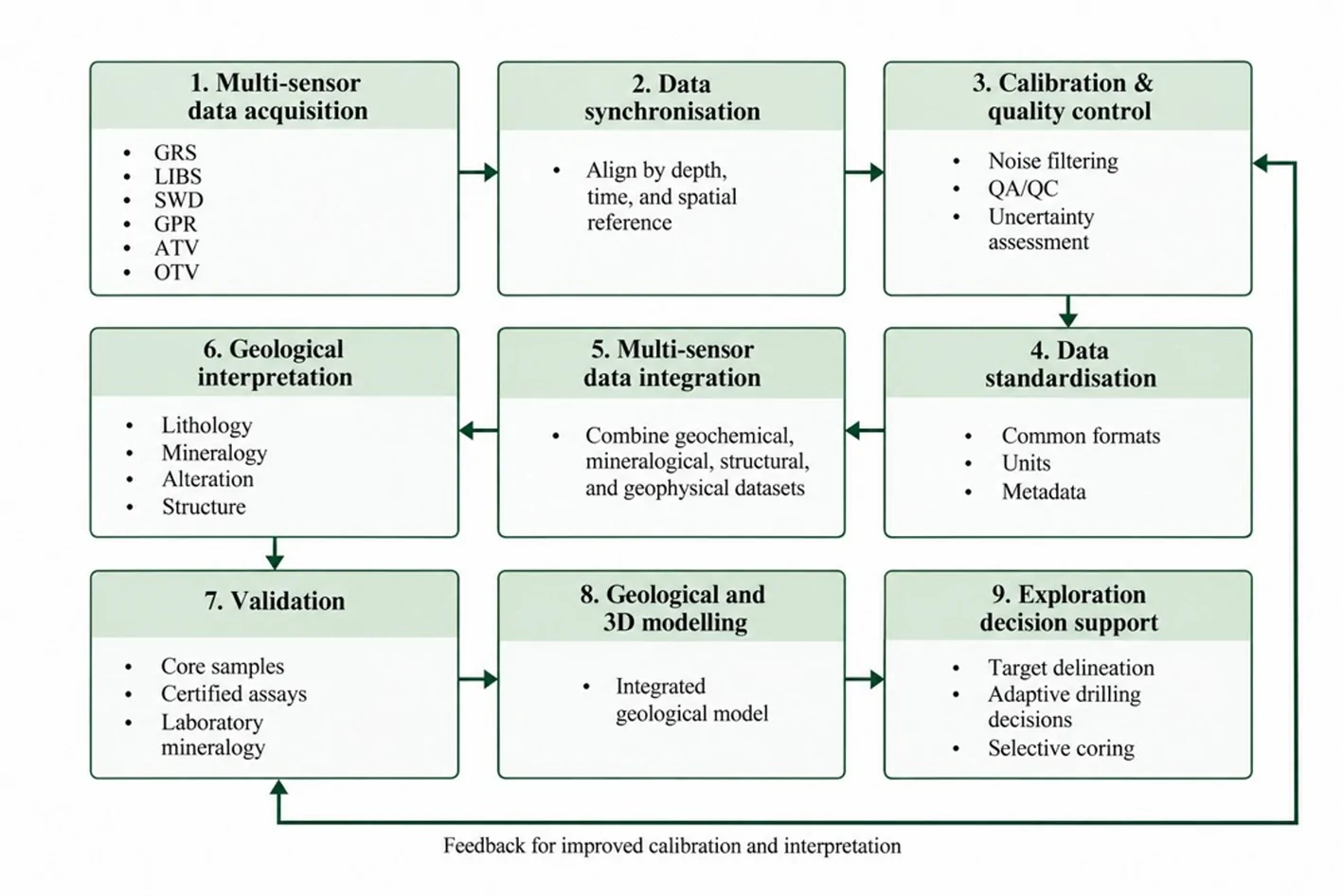
Proposed workflow for integrating multi-sensor downhole sensing data into geological modelling and exploration decision-making

A potential workflow would involve synchronizing data from individual sensors, applying sensor-specific calibration and quality control, and standardizing the resulting datasets. These processed datasets could then be integrated to generate a combined interpretation of lithology, mineralogy, geochemistry, and structure. The integrated data would then undergo validation against core samples, certified assays, laboratory mineralogical analyses, and other geological observations, as illustrated in Fig. 5. Finally, the validated data would be incorporated into geological models and decision-support workflows, where they could support target delineation, geological interpretation, adaptive drilling decisions, and prioritization of subsequent sampling or coring. This workflow would transform downhole sensing data from isolated sensor outputs into an integrated decision-support system. Achieving this will require substantial engineering advancements to move technologies currently at TRL 5–6 toward reliable while-drilling deployment. Key developments include the miniaturization of sensors to integrate into the bottom hole assembly, enhanced ruggedization to endure high-pressure, high-temperature, and vibration-intensive conditions, and the creation of robust telemetry systems capable of transmitting large volumes of data in real time. Furthermore, progress in real-time data processing and noise filtering, potentially aided by machine learning, will be crucial for ensuring data reliability and usability during drilling operations.

## Benefits to the mining industry

Downhole sensing while drilling could produce significant advantages for mineral exploration by providing geological information in real time and enhancing operational efficiency through integrated drilling systems. These technologies can complement traditional diamond drilling and core logging by offering structural, mineralogical, and geochemical data directly from the borehole. This integration can optimize drilling programs, improve field decision-making, and increase overall cost-effectiveness. By decreasing reliance on coring, companies could limit core retrieval to select holes, thereby reducing drilling and exploration campaign expenses and allowing rigs to be more flexibly deployed to different sites. Access to real-time subsurface data would accelerate decision-making as drill spacing could be adjusted instantly to capture missed features or minimize unnecessary drilling, resulting in more effective targeting. Insights into the geological information obtained during drilling would improve orebody delineation and refine geological models at earlier stages. This method could significantly shorten the overall exploration timeline, as traditional diamond core drilling often requires weeks to complete, whereas sensor-based drilling can produce information much more quickly. Additionally, downhole sensing could decrease the environmental impact of exploration by reducing energy consumption, and the need to handle, transport, and store large volumes of core. Furthermore, the integration of these sensors would create standardized digital datasets that minimize subjectivity in core logging, provide geologists with reliable references, and facilitate continuous integration into advanced modelling workflows and digital twins. The commercial advantages of real-time downhole sensing are significant. With the frequent fluctuations in commodity prices, the ability to quickly identify ore bodies, grades, and geomechanical properties allows for faster decision-making, earlier resource development, and a quicker return on investment. Additionally, by facilitating more targeted mining, these technologies can help minimize unnecessary excavation, especially if selective extraction becomes a viable outcome of their use.

## Conclusion

For decades, core drilling has underpinned geological exploration and has been widely regarded as highly reliable due to its ability to provide direct physical evidence of the subsurface. However, as exploration increasingly targets deeper and more complex ore bodies, conventional coring methods are becoming more time-consuming, expensive, and resource intensive. In this context, emerging non-invasive sensor technologies are increasingly capable of delivering comparable geological information with improved efficiency and/or greater spatial resolution. This presents an opportunity for the industry to rethink mineral exploration, making it more reliable, faster, adaptable, cost-effective, and data driven. Yet, given their current technology readiness level, downhole sensing during drilling should not currently be viewed as an imminent replacement for core drilling but instead as a complementary innovation that enhances the acquisition, interpretation, and application of geological information. Within this framework, the role of downhole sensing can be divided into three key application areas: (1) Enhanced spatial and geological understanding and data standardisation. Sensor-based systems such as XRF, LIBS, and GRS enable greater spatial and geological consistency by producing standardised, repeatable datasets that reduce interpretative subjectivity inherent in traditional core logging, while also extending geological understanding beyond discrete samples to more continuous subsurface characterisation. (2) Predictive subsurface capability. Forward-looking geophysical methods such as SWD, GPR, and BTEM introduce predictive capability by detecting lithological or mineralisation boundaries ahead of the drill bit, providing subsurface information that cannot be obtained from core, which is inherently retrospective. (3) Real-time decision-making and selective core targeting. Downhole sensing enables real-time, closed-loop geological decision-making during drilling, with systems such as SWD, GRS, and BTEM providing immediate lithological or structural feedback that allows drilling to be dynamically adjusted and enables core collection to be selectively targeted to high-value or high-uncertainty intervals, reducing unnecessary drilling through homogeneous or unmineralized units.

In the near term, technologies like GRS, SWD, and electromagnetic methods, including GPR and BTEM, XRF, ATV, and NbGT systems, are well-suited for real-time deployment due to their high level of technological readiness. In contrast, optical and imaging-based techniques such as HSI, LiDAR, and OTV represent longer-term opportunities due to challenges related to borehole conditions and system integration. A significant gap in current literature is the absence of fully integrated, real-time downhole sensing systems specifically designed for mineral exploration. Despite extensive research into individual sensing technologies, their combined deployment and validation in operational mineral exploration drilling environments remain largely unstudied. A further challenge is data interoperability; different sensors produce datasets with varying formats, spatial and temporal resolutions, depth references, and calibration requirements, making their integration into a common geological framework difficult. Future research should prioritize common data standards and interoperable workflows. These should be supported by large-scale field demonstrations and comparative studies to quantify how these technologies influence exploration outcomes, drilling efficiency, and resource discovery. Bridging this gap will require dedicated test mine facilities to support technology validation and integration. Such demonstrations are essential to build confidence among industry stakeholders, regulators, and investors. The knowledge gained from these demonstrations must then inform policy development, enabling the adoption of these technologies within reporting frameworks such as JORC and NI 43-101. Over the long term, this approach has the potential to herald a new paradigm in mineral exploration, where subsurface ore can be characterized substantially more efficiently than currently possible.

## Data Availability

All data supporting the findings of this study are available within the paper and its Supplementary Information.
